# A Novel Null Mutation in P450 Aromatase Gene (CYP19A1) Associated with Development of Hypoplastic Ovaries in Humans

**DOI:** 10.4274/jcrpe.2761

**Published:** 2016-06-06

**Authors:** Sema Akçurin, Doğa Türkkahraman, Woo-Young Kim, Erdem Durmaz, Jae-Gook Shin, Su-Jun Lee

**Affiliations:** 1 Akdeniz University Faculty of Medicine Hospital, Department of Pediatric Endocrinology, Antalya, Turkey; 2 Antalya Training and Research Hospital, Clinic of Pediatric Endocrinology, Antalya, Turkey; 3 Inje University College of Medicine, Department of Pharmacology, Inje University, Busan, Korea; 4 İzmir University Faculty of Medicine, Medical Park Hospital, Clinic of Pediatric Endocrinology, İzmir, Turkey

**Keywords:** Aromatase, CYP19A1 gene, ovarian development

## Abstract

**Objective:**

The CYP19A1 gene product aromatase is responsible for estrogen synthesis and androgen/estrogen equilibrium in many tissues, particularly in the placenta and gonads. Aromatase deficiency can cause various clinical phenotypes resulting from excessive androgen accumulation and insufficient estrogen synthesis during the pre- and postnatal periods. In this study, our aim was to determine the clinical characteristics and CYP19A1 mutations in three patients from a large Turkish pedigree.

**Methods:**

The cases were the newborns referred to our clinic for clitoromegaly and labial fusion. Virilizing signs such as severe acne formation, voice deepening, and clitoromegaly were noted in the mothers during pregnancy. Preliminary diagnosis was aromatase deficiency. Therefore, direct DNA sequencing of CYP19A1 was performed in samples from parents (n=5) and patients (n=3).

**Results:**

In all patients, a novel homozygous insertion mutation in the fifth exon (568insC) was found to cause a frameshift in the open reading frame and to truncate the protein prior to the heme-binding region which is crucial for enzymatic activity. The parents were found to be heterozygous for this mutation. Additionally, all patients had hypoplastic ovaries instead of cystic and enlarged ovaries.

**Conclusion:**

A novel 568C insertion mutation in CYP19A1 can lead to severe aromatase deficiency. Homozygosity for this mutation is associated with the development of hypoplastic ovaries. This finding provides an important genetic marker for understanding the physiological function of aromatase in fetal ovarian development.

**WHAT IS ALREADY KNOWN ON THIS TOPIC?**

Aromatase deficiency can cause various clinical phenotypes resulting from excessive androgen accumulation and insufficient estrogen synthesis. Aromatase deficiency has been reported to cause delayed puberty in adolescent girls, minimal or absent breast development, primary amenorrhea, hypergonadotropic hypogonadism, tall stature, delayed bone age, and enlarged and multicystic ovaries.

**WHAT THIS STUDY ADDS?**

A novel insertion mutation in the aromatase gene (CYP19A1) was found which caused a frameshift in the open reading frame and a truncation of the protein prior to the heme-binding region. Homozygosity for this mutation was associated with the development of hypoplastic ovaries. This finding provides an important genetic marker for understanding the physiological function of aromatase in fetal ovarian development.

## INTRODUCTION

Aromatase (CYP19A1) catalyzes the conversion of androgens to estrogens, which is a key step in estrogen biosynthesis (1,2). The enzyme is mainly located in the endoplasmic reticulum of estrogen-producing cells in the ovary, placenta, testis, brain, adipose tissue, liver, muscle, and hair follicles (3,4). During pregnancy, dehydroepiandrosterone sulfate (DHEAS) and 16OH-DHEAS, arising from the fetal adrenal gland and liver, respectively, become important sources for the synthesis of placental estrogens (4,5). To date, more than eleven genetic mutations in CYP19A1 gene have been reported in multiple studies with in vivo phenotype information (6,7,8,9,10,11,12,13,14). Placental aromatization of androgens has been suggested to be essential for protecting both the mother and any female fetus against the virilizing action of fetal androgens, particularly during differentiation of external genitalia (4). Autosomal recessive mutations in the CYP19A1 gene have been reported to cause disorders of sex development (DSD) and virilization of the mother during pregnancy (11,12,13,15). Here, we report the clinical and genetic features of three cousins with a novel homozygous mutation (568insC) in CYP19A1 that caused severe aromatase deficiency. The present study suggests that severe aromatase deficiency appears to be associated with hypoplastic rather than enlarged and multicystic ovaries.

## METHODS

### Study Subjects

**Figure 1:**
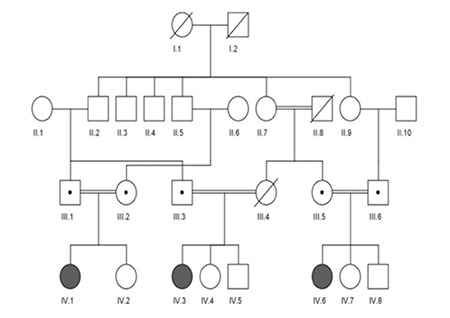
Pedigree of the family covering four generations. The symptomatic subjects with homozygous CYP19A1 mutation of a novel mutation (568insC) in exon 5 of CYP19A1 gene are shown as solid symbols. The slashed symbols represent deceased family members. Dots in circles refer to subjects carrying the heterozygous mutation. Circles represent female family members and squares male family members

Case 1 (index case, IV.3) was a Turkish female presenting with clitoromegaly and partial labial fusion at birth (Prader II), the third child of consanguineous parents (Figure 1). A history of voice changes and hirsutism in the mother during pregnancy was reported. The mother was also reported to demonstrate symptoms of hemolysis, elevated liver enzymes, low platelet count (HELLP) syndrome, and she died during labor. The infant was then hospitalized due to hypoxic-ischemic encephalopathy (HIE) and neonatal sepsis. Laboratory evaluation revealed hyperandrogenism without adrenal insufficiency. The karyotype was 46,XX. After discharge from the hospital, she was again admitted at 7 years of age. At this time, the girl was found to have severe cerebral palsy and failure to thrive [height: 103 cm, -3.6 standard deviation score (SDS); weight: 13 kg, -1.7 SDS]. She had no breast or pubic hair development but was noted to have clitoromegaly (1.5 cm). Pelvic ultrasonography (US) revealed a 26-mm uterus with hypoplastic ovaries (0.02 mL). Laboratory evaluation revealed normal androgen and basal gonadotropin levels with undetectable estradiol (Table 1).

Case 2 (IV.6) was a Turkish female presenting with clitoromegaly, complete labial fusion, and single urogenital sinus at birth (Prader III). She was a cousin of case 1 and the third child of consanguineous parents (Figure 1). A history of maternal voice changes, acne formation, and clitoromegaly during pregnancy was reported. Laboratory evaluation revealed hyperandrogenism without adrenal insufficiency. The karyotype was 46,XX. The patient underwent vaginoplasty. At her last visit, at 5 years of age, she had mild clitoromegaly (1 cm) without any other clinical signs of hyperandrogenism. Pelvic US revealed a 23-mm uterus with hypoplastic ovaries (0.06 mL). Laboratory evaluation revealed normal androgen and high basal gonadotropin levels with undetectable estradiol (Table 1).

Case 3 (IV.1) was a cousin of case 2 and presented with clitoromegaly, complete labial fusion, and single urogenital sinus at birth (Prader III) (Figure 1). A history of maternal voice changes and acne formation during pregnancy was reported. Laboratory evaluation revealed hyperandrogenism without adrenal insufficiency. The karyotype was 46,XX. Vaginoplasty was performed. At her last visit, at 2.2 years of age, she had clitoromegaly (1.5 cm) without any other clinical sign of hyperandrogenism. Pelvic magnetic resonance imaging revealed a rudimentary uterus (11 mm) with hypoplastic ovaries (0.04 mL). Laboratory evaluation revealed normal androgen and high basal gonadotropin levels with normal estradiol (Table 1).

The subjects were interviewed carefully to determine their lineage and birth and were considered to belong to a pedigree. After explanation of the study, written informed consent was obtained from all participants before obtaining blood samples. DNA could not be isolated from other siblings due to socio-economic reasons and parental non-compliance. However, according to the parents, those female siblings were healthy and had no clitoromegaly.

### Sequencing and Identification of CYP19A1 Variants

Genomic DNA was isolated from peripheral whole blood using a QiAamp blood kit (Qiagen, Valencia, CA, USA). The research protocol for the use of human DNA was approved by the institutional review board of Busan Paik Hospital, Busan, Korea, and conformed to institutional guidelines (16). The exons, intron-exon junctions, promoter region, and 3’-untranslated region of CYP19A1 were polymerase chain reaction (PCR)-amplified and directly sequenced. Primers for PCR amplification and DNA sequencing were identical to those used previously (16).

### Genetic Analysis of CYP19A1 Variants

Haploview 4.2 population genetic analysis software (http://www.broad.mit.edu/mpg/haploview/) was used to analyze linkage disequilibrium (LD) and haplotype diversity. The sequence analysis programs NNSPLICE 0.9 (www.fruitfly.org/seq_tools/splice.html) and TFSEARCH (www.cbrc.jp/research/db/TFSEARCH.html) were used to predict alternative splice sites and transcription factor-binding changes introduced by mutations, respectively.

## RESULTS

**Figure 2:**
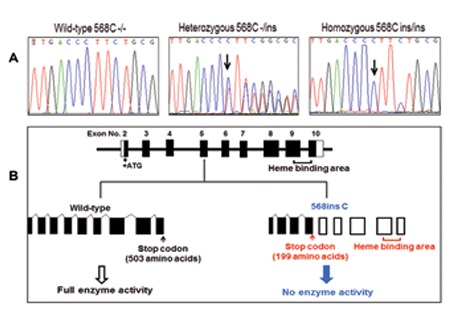
Aromatase deficiency resulting from a novel null mutation in the CYP19A1 gene. (A) DNA sequence of the CYP19A1 gene around the site of mutation in exon 5. The C CYP19A1 is indicated by the arrow. (B) Schematic representation of CYP19A1 gene structure and the location of the mutation of 568insC in the map. Black bars represent exons. Truncated CYP19A1 protein caused by the 568insC is indicated by the arrow, resulting in 199 amino acids

Direct DNA sequencing analysis of the CYP19A1 gene in all subjects (n=8) revealed a total of 12 genetic variants. A summary of the identified variants is presented in Table 2. Among these 12 variations, a 568Cins in exon 5 was previously unidentified. The 568Cins was found as a heterozygous mutation in parents and a homozygous mutation in the three probands (Figure 2A). This insertion mutation caused a change of amino acid 190Leu to 190Pro and the subsequent frameshift was predicted to generate a stop codon, resulting in a truncated protein of 199 amino acids rather than the full functional 503 amino acids of the CYP19A1 protein (Figure 2B). No particular linkage was found with this novel mutation. None of variants were predicted to create or disrupt splice sites or transcription factor-binding elements.

## DISCUSSION

Aromatase deficiency causes virilization of the mother during pregnancy and ambiguous genitalia of the female fetus due to prenatal exposure to adrenal androgens. In the postpartum period, some clinical features of androgen excess regress and elevated androgen concentrations return to normal levels. Aromatase deficiency has been reported to cause delayed puberty in adolescent girls, minimal or absent breast development, primary amenorrhea, hypergonadotropic hypogonadism, tall stature, delayed bone age, decreased bone mineral density, and multicystic ovaries (8,9). Additionally, it has been speculated that in aromatase-deficient prepubertal girls, an amplification of follicle-stimulating hormone (FSH) signaling might occur in the presence of high intraovarian androgen production and be responsible for the development of ovarian follicular cysts (4). However, affected male infants have normal internal and external genital development. Affected males have usually been diagnosed after puberty with tall stature, delayed bone age, decreased bone mineral density, and infertility (14). Interestingly, in our cases, the ovaries were hypoplastic without cyst formation. Luteinizing hormone (LH) and especially FSH levels were high at the final visits in cases 2 and 3 but not in case 1. Prepubertal levels of LH and FSH in case 1 may have been due to hypogonadotropic hypogonadism resulting from severe HIE during the neonatal period. Hypoplastic ovaries rather than enlarged ovaries in aromatase-deficient females have rarely been reported. There are only two cases with similar phenotype reported in the literature. Lin et al (12) reported a case of aromatase deficiency with hypoplastic ovaries and uterus. Karyotype was 46,XX. The patient had a severe mutation (exon5del) in exon 5 of the CYP19A1 gene, generating a truncated protein without the heme-binding region crucial for enzymatic activity. Recently, another case of severe aromatase deficiency due to a 27-base duplication in exon 8 has been reported (17). The case was a 25-year-old woman with delayed puberty and osteoporosis. She had a hypoplastic uterus and bilateral streak ovaries with 46,XX karyotype. The authors concluded that the streak ovaries may be an inherent manifestation of CYP19A1 deficiency. These two cases, similar to the present cases, exhibited a severe phenotype with clitoromegaly, labial fusion, and/or single urogenital sinus at birth.

Indeed, as the studies of CYP19A1 knockout (ArKO) mouse model have shown, follicular development in ovaries of ArKO is abnormal in an age-dependent manner, with an early block in follicular development at the antral stage with absent corpora lutea. Then, haemorrhagic cysts with absent secondary and antral follicles and atresia of primary follicles with increased collagen deposition are observed. Additionally, in the ArKO mouse, uterine weight was found to be very low compared to that of wild-type, possibly because of hypoestrogenism (18). These findings are consistent with those reported in the CYP19A1 knockout mouse.

Taking into consideration all these reports, we speculate that severe aromatase deficiency in intrauterine life can cause insufficient production of fetal estrogens in the human fetus as well as testosterone excess which in turn might result in maldevelopment of the fetal ovaries. However, it is obvious that not all patients with severe aromatase deficiency have hypoplastic ovaries. This can be explained by the fact that gonadal development is a multifactorial process and involves many genes that interact with each other. Further in vitro and in vivo studies are necessary to understand this complex process.

In conclusion, a novel genetic variant in CYP19A1 gene was found to generate a null mutation. We suggest that severe aromatase deficiency caused by 568insC mutation can result in maldevelopment of ovaries in female fetuses. Further studies in large numbers of subjects would be necessary to understand the effect of this mutation on cancer, osteoporosis, and ovarian development.

## Ethics

Ethics Committee Approval: Institutional Review Board of Busan Paik Hospital, Informed Consent: It was taken.

Peer-review: Internal peer-reviewed.

## Figures and Tables

**Table 1:**
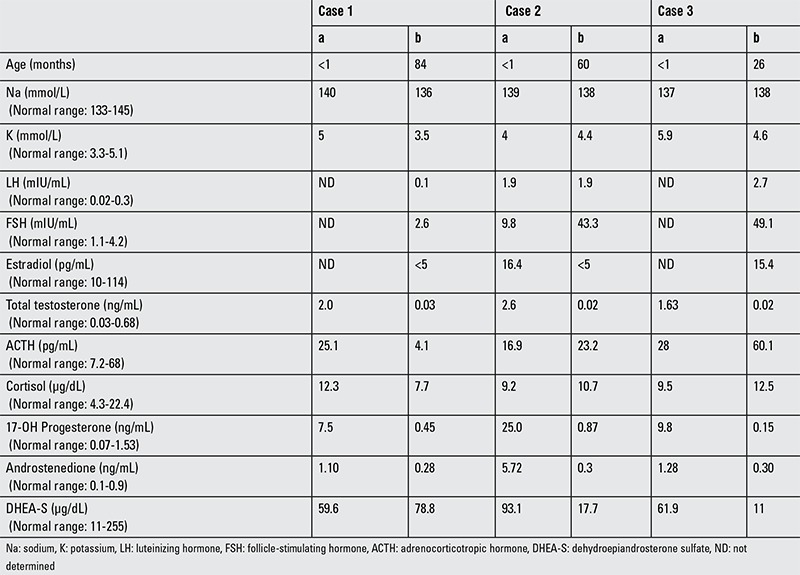
Laboratory values at diagnosisa and at last visit^b^

**Table 2:**
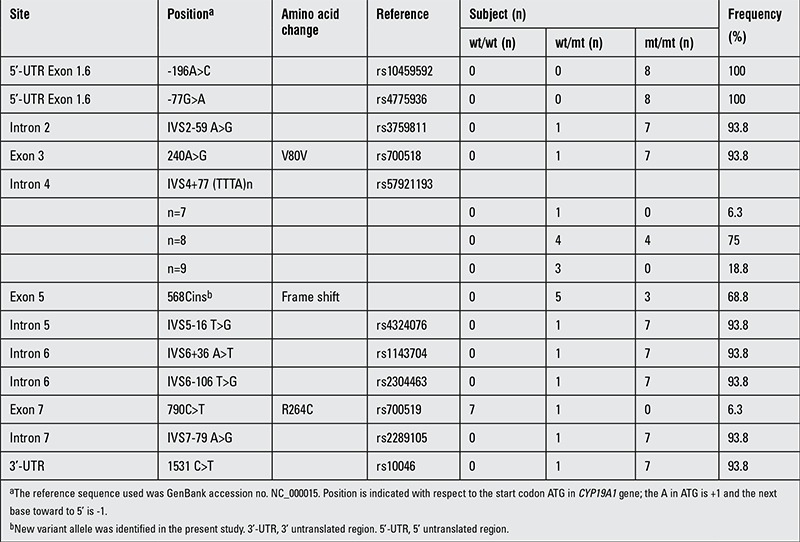
CYP19A1 single-nucleotide polymorphisms in the subjects (n=8)
